# A New Adaptive Entropy Portfolio Selection Model

**DOI:** 10.3390/e22090951

**Published:** 2020-08-28

**Authors:** Ruidi Song, Yue Chan

**Affiliations:** Institute for Advanced Study, Shenzhen University, Shenzhen 518060, Guangdong, China; flute68@163.com

**Keywords:** portfolio optimization, information entropy, risk measures, quantitative trading strategies, C61, G11

## Abstract

In this paper, we propose an adaptive entropy model (AEM), which incorporates the entropy measurement and the adaptability into the conventional Markowitz’s mean-variance model (MVM). We evaluate the performance of AEM, based on several portfolio performance indicators using the five-year Shanghai Stock Exchange 50 (SSE50) index constituent stocks data set. Our outcomes show, compared with the traditional portfolio selection model, that AEM tends to make our investments more decentralized and hence helps to neutralize unsystematic risks. Due to the existence of self-adaptation, AEM turns out to be more adaptable to market fluctuations and helps to maintain the balance between the decentralized and concentrated investments in order to meet investors’ expectations. Our model applies equally well to portfolio optimizations for other financial markets.

## 1. Introduction

Portfolio theory is an important theoretical tool for making a sound investment decision. Markowitz [1] used variance as a quantitative basis for risk measurement and proposed the mean-variance model for portfolio selections, based on the probability theory and goal programming methods. Its essence is to establish a model, which is suitable for various types of risk preference investors. He also proposed an effective methodology to obtain optimal portfolios. The appearance of such theories had pushed the development of finance forward from a qualitative stage into a quantitative stage, triggering a large amount of research on modern investment portfolios. On the other hand, the result of Bera and Park [2] observed that the portfolio weights, obtained from the mean-variance model, resulted in focusing on a few assets or extreme positions under extreme market situation, violating an important objective of diversification in asset allocations. As we all known, it is not wise to put all eggs in one basket. In other words, the more dispersed the weight of the portfolio, the lower the risk of the final portfolio [3,4]. Moreover, compared with investing in a few single assets, a diversified portfolio also has lower volatility [5].

So how to measure diversity? Actually, entropy is a widely accepted measure for diversity [6,7,8,9,10,11,12]. Shannon [13] proposed the concept of “information entropy”, which has been successfully applied in finance [11,12]. As we all know, as entropy increases, so does portfolio diversification. Early literature using entropy as an objective function for multi-objective model of portfolio selections include [2,8,9,12,14,15]. Philippatos and Wilson were the first two researchers who applied the concept of entropy to portfolio selection [16]. Bera and Park [2] presented asset allocation models, maximizing the entropy of the portfolio weight vector in order to generate a diversified portfolio. Jana et al. [15] added an entropy term into the mean-variance-skewness model, in order to cultivate more diversified asset portfolios upon optimizing the asset allocation. Using out-of-sample tests, Usta and Kantar [12] tested the mean-variance-skewness-entropy model with the entropy factor, resulting in much better performance than simply using Markowitz’s mean-variance model. Zhu and Cao [17] generalized their entropy model with a risk aversion index taking into account different types of risk investors and applied their model in Chinese securities market. Hence, in this study, we adopt the notation of variances [1] and entropy [13] to tackle the portfolio’s diversification.

Investment urgently requires an effective portfolio strategy to achieve high returns and low risk outputs. Therefore, it is vital to develop a portfolio theory, which is able to accommodate different market situations. In this article, we will propose a new adaptive entropy model (AEM), where “adaptive” denotes the adaptive change in the parameter λ (see Equation (12)) leading to a more decentralized investment under volatile market situations or a concentrated investment in certain high-quality stocks under soaring market situations. Moreover, we will adopt the five-year SSE50 index component data to validate our new model. Our results reveal that AEM can achieve higher returns while meeting the conditions of diversified investments that precedent models fail to make.

## 2. Entropy Model Description

### 2.1. Markowitz’s Mean-Variance Model

In this section, we will first introduce the Markowitz’s mean-variance model for our reference [1]. Suppose we select *n* risky assets. The vector of returns is $R={\left(r_{1},r_{2},\ldots ,r_{n}\right)}^{T}$, where $r_{i}$ represents the return of the *i*th risky asset and *T* means the usual transpose of a vector. Also, the vector of portfolio weights is $X={\left(x_{1},x_{2},\ldots ,x_{n}\right)}^{T}$, where $x_{i}$ is the weight of *i*th risky asset in the portfolio. There is no doubt that the portfolio weights hence satisfy the normalization condition. Additionally, each portfolio weights are constrained to be $x_{i}\in \left[0,1\right]$, where i=1,…,n, implying that any short selling is forbidden. The vector of excepted returns is $E\left[R\right]=M={\left(m_{1},m_{2},\ldots ,m_{n}\right)}^{T}$, where $m_{i}=E\left(r_{i}\right)$ and *E* denotes the expectation operator. Moreover, the n×n covariance matrix of returns is $V=E{\left[R-E\left[R\right]\right]}^{2}$.

The equations for calculating the mean and variance of portfolio returns are given, respectively as follows:

(1) $$E\left[r_{p}\right]=E\left[X^{T}R\right]=X^{T}M,$$

where $r_{p}=\sum_{i=1}^{n}x_{i}r_{i}$ is the return of portfolio. Variance of portfolio return reads

(2) $$\sigma^{2}\left[r_{p}\right]=E{\left[X^{T}R-E\left[X^{T}R\right]\right]}^{2}=X^{T}VX$$

Therefore, Markowitz’s mean-variance model can be written as follows:

(3) $$\begin{array}{cc} \; & \min\;\;X^{T}\mathrm{VX}-X^{T}M\\ \; & s.t.\;X^{T}1=1\;\left(0\leq x_{i}<1,\;i=1,2,\cdots ,N\right). \end{array}$$

where 1 is a n×1 all-ones vector. The existence of a unique minimizer for Markowitz’s mean-variance model is guaranteed by Theorem 1.

**Theorem** **1.** *The objective function of Equation (3) is a convex function.*

**Proof** **of Theorem 1.** For function $f\left(X\right)=X^{T}\mathrm{VX}-X^{T}M$, we take $B={\left(b_{1},b_{2},\ldots ,b_{n}\right)}^{T}$ and $C={\left(c_{1},c_{2},\ldots ,c_{n}\right)}^{T}$ as two weight vector and make γ∈(0,1) be a constant. Then, we need to prove the following inequality for convexity:

(4) $$f\left(\gamma B+(1-\gamma )C\right)\leq \gamma f\left(B\right)+\left(1-\gamma \right)f\left(B\right),$$

which can be reduced into:

(5) $$\gamma^{2}B^{T}\mathrm{VB}+{\left(1-\gamma \right)}^{2}C^{T}\mathrm{VC}\leq \gamma B^{T}\mathrm{VB}+\left(1-\gamma \right)C^{T}\mathrm{VC}.$$

Finally, we obtain:

(6) $$\gamma \left(\gamma -1\right)\left(B^{T}\mathrm{VB}+C^{T}\mathrm{VC}\right)\leq 0,$$

where γ(γ−1) is less than zero because γ∈(0,1). Moreover, the covariance matrix V is symmetrically positive definite. That is to say, $(B^{T}\mathrm{VB}+C^{T}\mathrm{VC})\geq 0$. Hence, we prove the theorem, which paves the way for paving the existence and the uniqueness of our model as shown below. □

### 2.2. Information Entropy

Shannon initiated the concept of information entropy in conjunction with communication theory in 1948, which has been successfully used in decision science and finance [13]. Given an event, we can assume that the probability distributions are $P=(p_{1},p_{2},\cdots ,p_{n})$, which satisfy the normalization condition $\sum_{i=1}^{n}p_{i}=1\;\left(i=1,2,\cdots ,n\right)$. In addition, the probability distributions can be replaced by the vector of portfolio weights $X={\left(x_{1},x_{2},\cdots ,x_{n}\right)}^{T}$. The discrete version of the entropy function H(P) is given by:

(7) $$H\left(P\right)=-\sum_{i=1}^{n}p_{i}lnp_{i}.$$

The information entropy function has three mathematical characteristics, which can complement the mean-variance model:

- Non-negative: For $0\leq p_{i}\leq 1$, $lnp_{i}\leq 0$ and $-\sum_{i=1}^{n}p_{i}lnp_{i}\geq 0$, equality happens when one $p_{i}=1$, and the others are zero, H(P)=0. If the entropy is incorporated in an objective function to determine portfolio weights, the obtained weights automatically become non-negative. This implies that a model with entropy always yields no short-selling. Due to the theoretical and practical reasons, the portfolio with no short-selling is preferred by most conservative investors [15];
- Extremum property: It has its maximum value ln(n), when $x_{i}=1/n\;\mathrm{for}\;i=1,\ldots ,n$ and reaches the minimum value 0 when $x_{i}=1\;\mathrm{and}\;x_{j}=0,i\neq j\;\mathrm{for}\;j=1,\ldots ,n$. As a result, H(P) is a good measurement of portfolio diversification [2];
- Strictly concave: So that the critical points are exactly maximizers.

We put the process of proving the convexity of the entropy function in the Appendix A. It is easy to see that while H(P) is strictly concave down, −H(P) is strictly concave up. In general, when the objective function of convex programming is strictly convex, there exists a unique minimizer and this minimizer must be the only optimal solution, which is useful in maintaining the well-posedness of our model. It was known that the mean-variance model (MVM) is also strictly convex [1]. Hence, the linear combination of the MVM and −H(P) is also strictly convex. We comment that the return distribution might not follow exponential forms, q-entropy could make more accurate results, where we can fit the return distribution with a Tsallis-Pareto distribution to obtain the q-value subjecting to future scrutinization.

### 2.3. Adaptive Entropy Model

Assuming the same conditions as stated in Equations (1)–(3), Zhu and Cao proposed a new portfolio selection model, which based on the entropy concept [17]. The details of the model are provided as follows:

(8) $$\begin{array}{cc} \; & \min\;\;\lambda \left(X^{T}\mathrm{VX}-\xi H\left(X\right)\right)-X^{T}M\\ \; & s.t.\;X^{T}1=1\;\left(0\leq x_{i}<1,\;i=1,2,\cdots ,N\right), \end{array}$$

where λ,ξ are real constants. As we can see, the model attempts to maximize both the return and the entropy of the portfolio, but minimize the variance. In addition, the Equation (8) attains a unique minimum value, because of the symmetrically positive-definite of the covariance matrix and the concavity of the entropy function. However, the objective function is static, where λ,ξ are assumed to be constants, which is not adaptive enough for describing the dynamic market.

To make the portfolio more responsive to dynamic markets, we propose an adaptive model for a portfolio selection, which is given as follow:

(9) $$\begin{array}{cc} \; & \min\;\;X^{T}\mathrm{VX}-\lambda X^{T}M-\left(1-\lambda \right)\xi H\left(X\right)\\ \; & s.t.\;X^{T}1=1\;\left(0\leq x_{i}<1,\;i=1,2,\cdots ,N\right). \end{array}$$

Analogous to the previous model (see Equation (8)), ξ is also a fixed real constant, and we aim to maximize the return and the entropy of portfolio but minimize the variance. However, to the authors’ best knowledge, we comment that no existing literature considers such λ, where λ is a variable depending on the past market performance.

With the same condition as given in Equations (1)–(3), we take into account the element of time effects by varying λ. Suppose the portfolio weight vectors of the last step is $X_{t-1}={\left(x_{1,t-1},x_{2,t-1},\cdots ,x_{n,t-1}\right)}^{T}$, where $x_{i,t-1}$ denote the weight of *i*th risky asset in the previous (t−1) time step. In particular, we allocate one month for a time step. The return vectors of the previous step is $R_{t-1}={\left(r_{1,t-1},r_{2,t-1},\ldots ,r_{n,t-1}\right)}^{T}$, where $r_{i,t-1}$ represents the return on the *i*th risky asset in the (t−1) time step. The vectors of mean return is $E\left[R_{t-1}\right]=M_{t-1}={\left(m_{1,t-1},m_{2,t-1},\ldots ,m_{n,t-1}\right)}^{T}$, where $m_{i,t-1}=E\left(r_{i,t-1}\right)$. The n×n covariance matrix of the previous step is $V_{t-1}=E{\left[R_{t-1}-E\left[R_{t-1}\right]\right]}^{2}$. In addition, $\lambda_{t-1}$ and $\lambda_{t}$ refer to the λ value of the previous step and the current step, respectively. We propose (0,0.1,0.2,…,1) for the initial value of $\lambda_{0}$, and contemplate these adaptive values to form the following formula.

(10) $$\begin{array}{cc} \; & \min\;\;X_{t-1}^{T}V_{t-2}X_{t-1}-\lambda_{0}X_{t-1}^{T}M_{t-2}-\left(1-\lambda_{0}\right)\xi H\left(X_{t-1}\right)\\ \; & s.t.\;X_{t-1}^{T}1=1\;\left(0\leq x_{i,t-1}<1,\;i=1,2,\cdots ,N\right). \end{array}$$

Then, $\lambda_{t}$ can be obtained by

(11) $$\lambda_{t}=argmax\left(X_{t-1}^{T}M_{t-1}\right)$$

Finally, we incorporate $\lambda_{t}$ into Equation (9) to obtain

(12) $$\begin{array}{cc} \; & \min\;\;X_{t}^{T}V_{t-1}X_{t}-\lambda_{t}X_{t}^{T}M_{t-1}-\left(1-\lambda_{t}\right)\xi H\left(X_{t}\right)\\ \; & s.t.\;X_{t}^{T}1=1\;\left(0\leq x_{i,t}<1,\;i=1,2,\cdots ,N\right). \end{array}$$

We comment here that traditional models guide financial practices how to invest. In addition, those existing models based only on means and covariances, which can be obtained from history data. Our adaptive entropy model also considers the entropy (diversification) of the portfolio. In addition to these, we intend to add the adaptive parameter $\lambda_{t}$ to maintain the balance between the expected return and the diversification under various dynamical market situations, where the market factors can be absorbed in $\lambda_{t}$.

To evaluate such self-adaptive effects, here, we make a comparison model with the fixed value of λ=0.5, so that the entropy model without adaptive effects (EMWA), is shown below:

(13) $$\begin{array}{cc} \; & \min\;\;X^{T}\mathrm{VX}-\frac{X^{T}M}{2}-\frac{\xi H(X)}{2}\\ \; & s.t.\;X^{T}1=1\;\left(0\leq x_{i}<1,\;i=1,2,\cdots ,N\right). \end{array}$$

On the other hand, we also designed a control group to measure the utility of entropy in the model. We simply add adaptiveness to Markowitz’s model, which is called adaptive mean-variance model (AMVM), the model is as follows

(14) $$\begin{array}{cc} \; & \min\;\;\left(1-\lambda_{t}\right)X_{t}^{T}V_{t-1}X_{t}-\lambda_{t}X_{t}^{T}M_{t-1}\\ \; & s.t.\;X_{t}^{T}1=1\;\left(0\leq x_{i,t}<1,\;i=1,2,\cdots ,N\right), \end{array}$$

where the symbols are consistent with that in Equation (12).

## 3. Empirical Study

Backtesting is a process that a trader simulates a trading strategy using historical data to generate numerical results and analyze its risk and profitability, which is often performed before risking any actual capital. In this section, we will carry out the evaluation of backtesting portfolios, and present the backtesting result.

### 3.1. Portfolio Performance Evaluation

Hence, we provide some common backtesting performance indicators, which will be used in the later subsections. Traditionally, The Capital Asset Pricing Model (CAPM) reveals the relationship between systematic risk and the excess return for assets or portfolios, which is widely used throughout the finance for pricing risky securities. The formula for calculating the expected return of a portfolio incorporating its risk is given as follow:

(15) $$E\left(r_{p}\right)=r_{f}+\beta \left(E\left(r_{m}\right)-r_{f}\right),$$

where $E(r_{p})$ and $E(r_{m})$ represent the expected return of portfolio and market (index), respectively, $r_{f}$ represents the risk-free rate, and β refer to the beta of the portfolio. Given that, we can derive two indicators: β and α, β represents the systematic risk, while α means the excess return of portfolio. Firstly, β can be calculated using this equation,

(16) $$\beta =\frac{Cov(r_{p},r_{m})}{\sigma^{2}\left[r_{m}\right]}.$$

On the other hand, the formula for calculating α is given as follows:

(17) $$\alpha =E\left(r_{p}\right)-r_{f}-\beta \left(E\left(r_{m}\right)-r_{f}\right).$$

As a traditional performance measure, the Sharpe ratio has been extensively used in stock markets, which is given by:

(18) $$\mathrm{Sharpe}\;\mathrm{Ratio}=\frac{E\left[r_{p}\right]}{\sqrt{\sigma^{2}\left[r_{p}\right]}},$$

where $r_{p}$ is the return of portfolio.

However, the Sharpe ratio only considers the mean and the volatility of the portfolio. An investor must take the worst situation into account, so there exists an indicator measuring the drawdown of the portfolio, namely the maximum drawdown (MDD). MDD taking up to the time *T* can be calculated using the following equation:

(19) $$\mathrm{Maximum}\;\mathrm{Drawdown}(T)=\max_{\tau \in (0,T)}\left(\max_{t\in (0,\tau )}\frac{VP_{t}-VP_{m}}{VP_{m}}\right),$$

where $VP_{t}$ represents the value of a portfolio at the time *t* and $VP_{m}$ denotes the maximum value in the time range, t∈(0,τ). Furthermore, Young proposed an indicator based on the MDD in 1991 [18], which is the calmar ratio:

(20) $$\mathrm{Calmar}\;\mathrm{Ratio}=\frac{E\left[r_{p}\right]}{\mathrm{MDD}}.$$

Furthermore, the win rate is an indicator used to measure the effectiveness of an investment strategy, which is the percentage of profitable trades for a given backtesting run. Win rate can be calculated by using:

(21) $$\mathrm{Win}\;\mathrm{Rate}=\frac{N_{win}}{N_{trade}},$$

where $N_{win}$ and $N_{trade}$ are the number of wins and trades in a backtesting run, respectively.

### 3.2. Results of the Empirical Study

We use MYQUANT (www.myquant.cn) as a backtesting platform and use the SSE50 index constituents from January 2014 to December 2018 as our stock pool. It is worth noting that the SSE50 index is only used to verify the model, and the model is also applicable to other stock markets. In order to test the adaptability of our proposed portfolio strategy for different Chinese market environments, we evaluate the performance of our strategy for each year. It should be emphasized here that all datasets are adjusted for capital splits and stock dividends.

At the time of backtesting, we set the beginning of each month as the first trading day to buy and hold stocks for a month duration, then we reconstruct the portfolio on the first trading day of the next month. In order to make the added and subtracted values on the same level, we found that it is more appropriate to set the ξ value in Equations (12) and (13) to be 0.0001. We also use the data of the last 30 daily bars to compute the means and standard deviations in Equations (1) and (2). For each year of backtesting, we will compare four separate strategies, namely Markowitz’s mean-variance model, MVM (see Equation (3)), entropy model without adaptive effects, EMWA (see Equation (13)), and adaptive entropy model, AEM (see Equation (12)), adaptive mean-variance model, AMVM (see Equation (14)) in order to shine a light on our adaptive methodology, where the numerical results are shown in Table 1.

### 3.3. Results and Analysis

It can be seen from Table 1 that the β values for our AEM are similar to that of EMWA and MVM. However, AEM has larger α values than that of EMWA and the comparable α values to that of MVM, which is a promising outcome implying that all portfolio models have the similar systematic risks. In other words, AEM has higher excess return than EMWA and a comparable return to MVM. It must be mentioned that the AMVM performance was extremely unstable, and even had a negative alpha in 2014. Such outcomes are due to the fact that the λ values control the centralization and decentralization of our portfolio by aligning the trend between the portfolio and the index. Moreover, AEM uses the adoption of λ values to outperform the index and other static variance models, thereby meeting our common objectives of high returns and the diversification. In addition, AEM including the three control strategies performs better than the benchmark except in 2014. That is to say that our proposed investment strategy is in general more advanced than simply buying an index. It is worthy mentioning that the maximum drawdown of our AEM is consistently outperformed that of the MVM and AMVM, presumably because we have incorporated the measurement of entropy. Furthermore, we have obtained the return series of AEM and MVM in 2015 and performed a one-sided test to verify whether the return of AEM is significantly higher than that of MVM. The final statistic and the P value are 1.412 and 0.0796, respectively, i.e., at a significance level of 10%. Accordingly, we can infer that the rate of return of AEM is higher than that of MVM. Coincidentally, Chinese stock market experienced a crash in 2015. Therefore, our AEM appears to have the stronger adaptability for responding to tumbling markets. From the backtest results from 2014 to 2018, the returns and anti-risk capabilities of AEM is significantly better than other control strategies. Considering adaptive or entropy alone cannot construct a good portfolio but AEM considers both.

**Figure 1:**
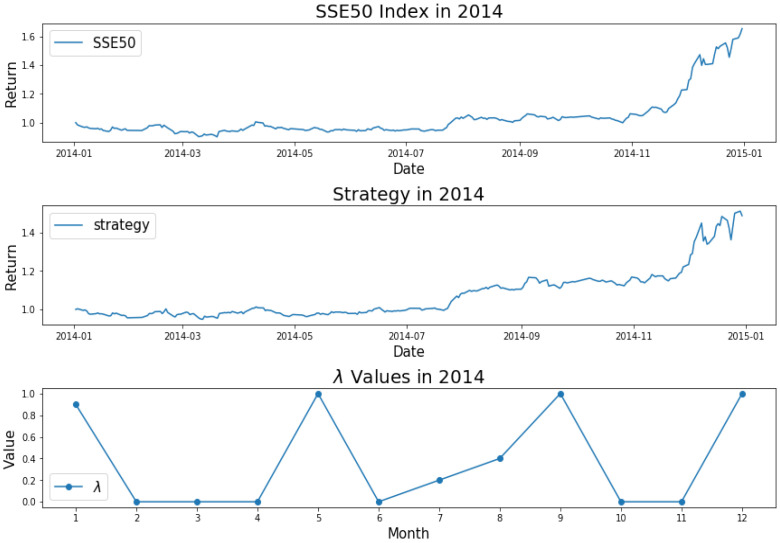
SSE50 Index in 2014 (**upper**); strategy in 2014 (**middle**); λ Values in 2014 (**lower**).

**Figure 2:**
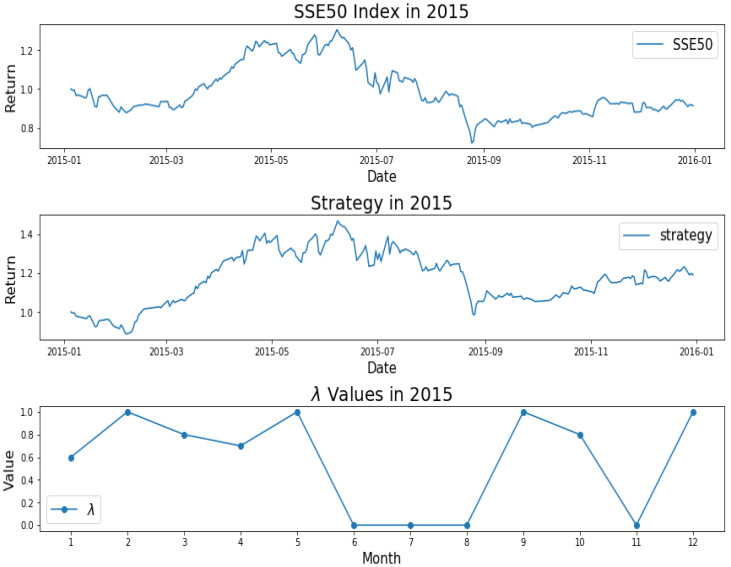
SSE50 Index in 2015 (**upper**); strategy in 2015 (**middle**); λ Values in 2015 (**lower**).

**Figure 3:**
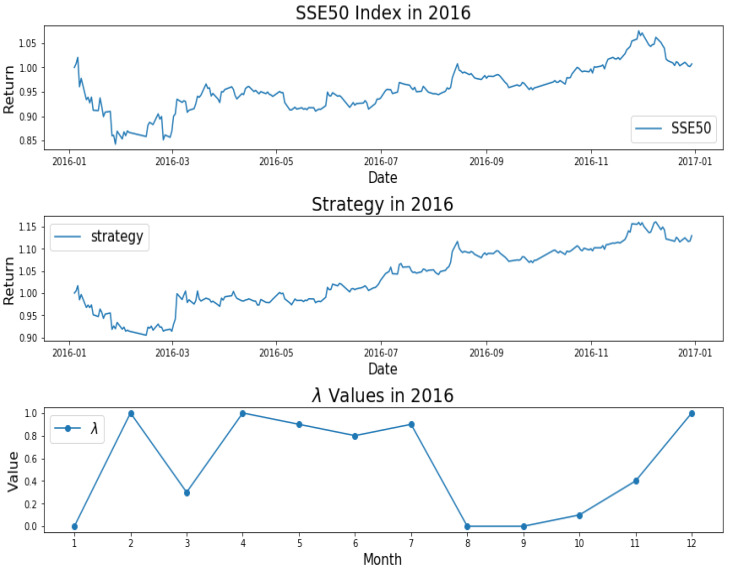
SSE50 Index in 2016 (**upper**); strategy in 2016 (**middle**); λ Values in 2016 (**lower**).

**Figure 4:**
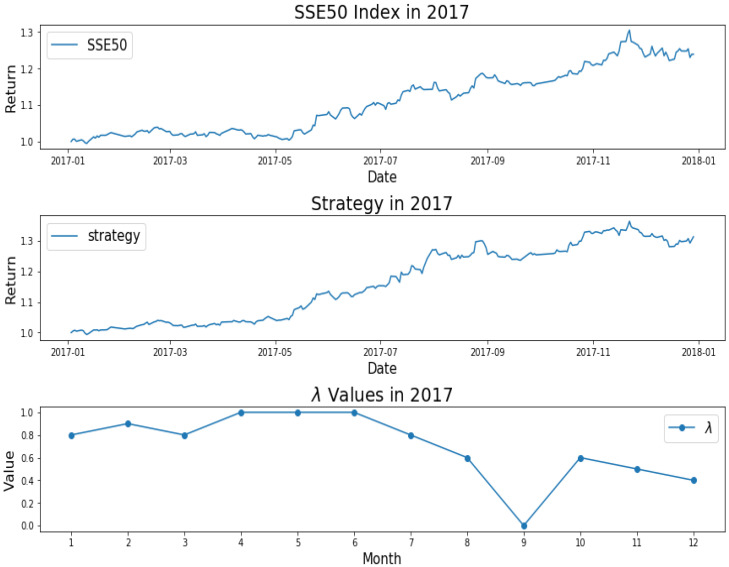
SSE50 Index in 2017 (**upper**); strategy in 2017 (**middle**); λ Values in 2017 (**lower**).

**Figure 5:**
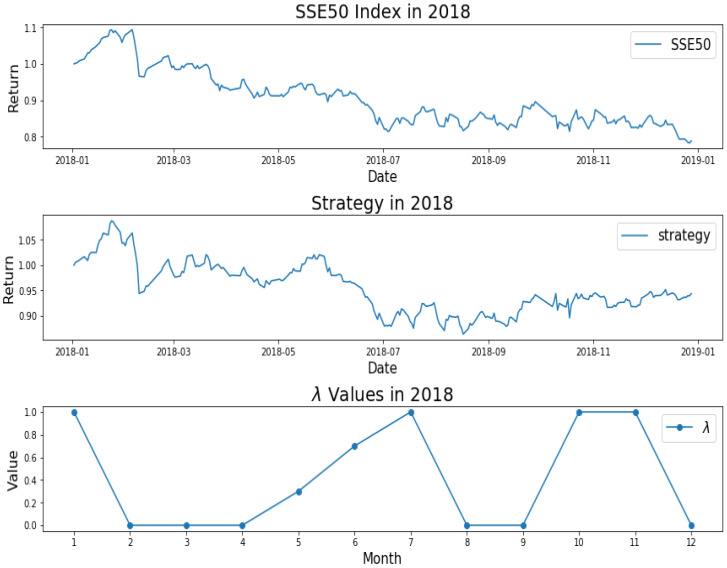
SSE50 Index in 2018 (**upper**); strategy in 2018 (**middle**); λ Values in 2018 (**lower**).

How do we keep the balance between return and decentralized investment? To better investigate the mechanism of AEM, we plot the changes of λ values over time, as shown from Figure 1, Figure 2, Figure 3, Figure 4 and Figure 5. Evidently, we can observe the working mechanism of the adaptive entropy model that the closer the λ value is to zero, the more diversified the investment. On the contrary, the closer the λ value is to one, the more concentrated the investment. These values also coincide with the trend of the SSE50 index, for example as given in Figure 2.

**Figure 6:**
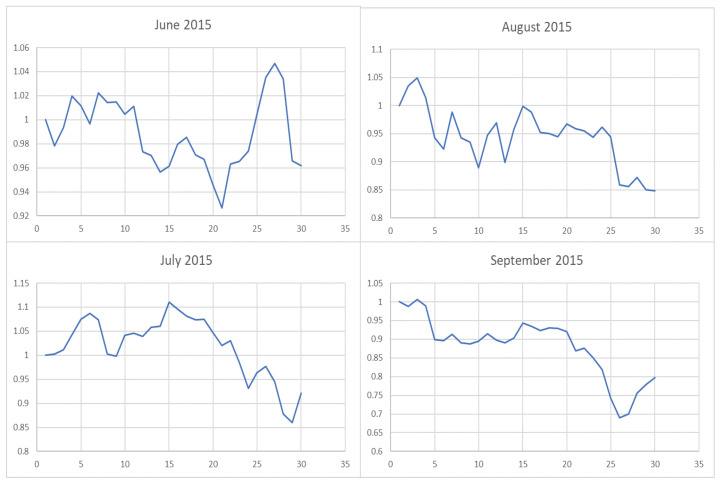
The trend of the index (market conditions) when calculating the λ value from June to September in 2015.

**Figure 7:**
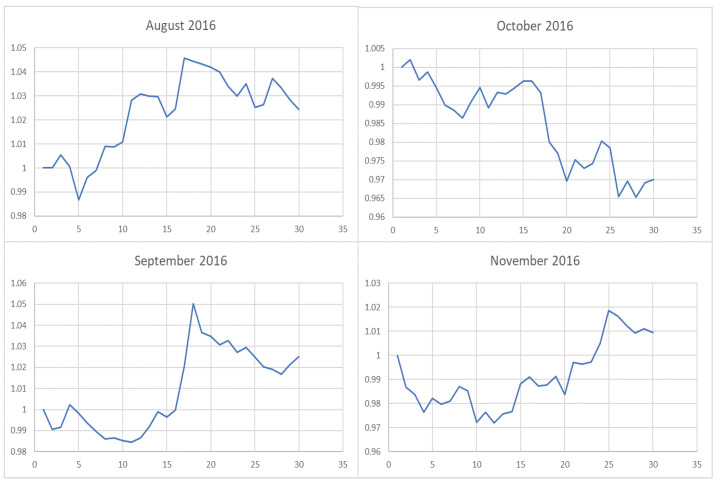
The trend of the index (market conditions) when calculating the λ value from August to November in 2016.

**Figure 8:**
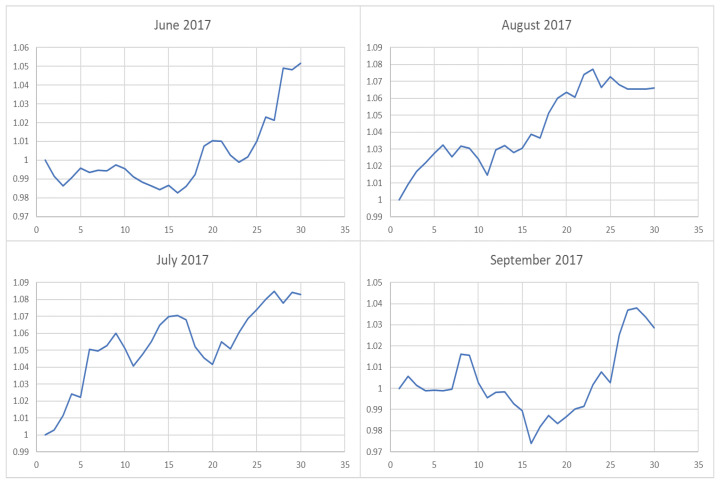
The trend of the index (market conditions) when calculating the λ value from June to September in 2017.

**Figure 9:**
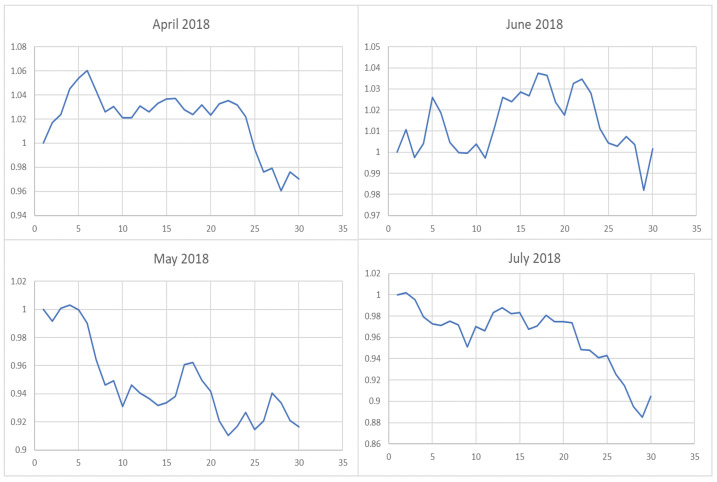
The trend of the index (market conditions) when calculating the λ value from April to July in 2018.

However, it is obvious that sometimes, the trend of the value of λ will be opposite to the trend of the index. For example, June to September in 2017 and April to July in 2018. The reason behind this is as follows: Actually, the λ value of the current cycle depends on the data of previous 30 trading days. In order to standardize the process, the cycle is not divided by the natural months. Hence, the λ value will generally correlate with the trend of an index, but we also need to take into account the situation of individual stocks and the various states of the market. Accordingly, we analyze the data from April to August in 2015, June to October in 2016, April to August in 2017, and February to August in 2018. In order to illustrate why the trend of the index and the λ is opposite, we plot their trend in Figure 6, Figure 7, Figure 8 and Figure 9.

We found that when the market is in a state of unilateral rise or decline, the λ value approaches 1 and therefore the investment is concentrated in a few high-quality stocks resulting in excess returns, such as in 2017/06, 2017/07, while in the market downturn, high-quality stocks will be more risk-resistant and their prices will tend not to fall as the index falls, such as in 2015/09 and 2018/07. On the contrary, if the index fluctuates greatly, such as during a deep V pattern, the model accommodate the response by diversifying the investment. Likewise, the rapid rotation of the plate, and the dramatically rise and fall will make us unwise to invest in a few stocks, such as in 2015/06, 2016/09, 2017/09, 2018/04.

**Figure 10:**
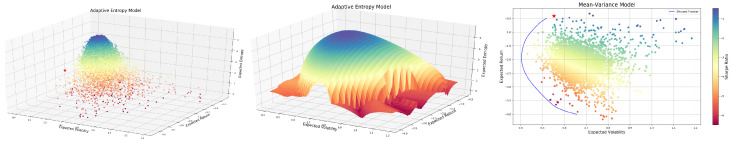
Model Comparison in August 2015 using Frontier Figure.

**Figure 11:**
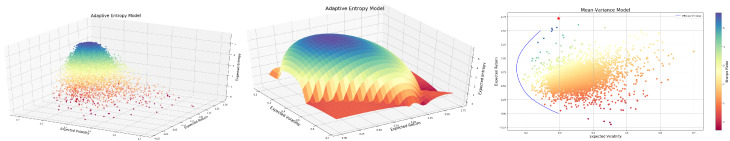
Model Comparison in March 2016 using Frontier Figure.

**Figure 12:**
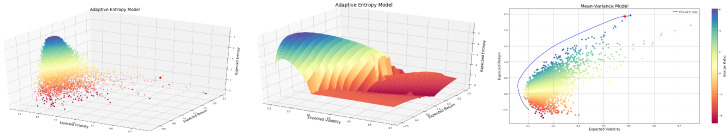
Model Comparison in August 2017 using Frontier Figure.

Since AEM and MVM have comparable excess returns, we will visualize the differences between MVM and AEM in the perspective of entropy. We have selected market data in August 2015, March 2016 and August 2017, which correspond to the decline, the rise, and the sideways market conditions, respectively to make such comparison. We drew effective frontier figures and plot them in three-dimensional figures as shown in Figure 10, Figure 11 and Figure 12. It can be easily observed for AEM results that when the entropy of the investment portfolio increases, the investment will be more dispersed, so that the returns and risks will be more moderate, which is the reason it is not wise to put all eggs in one basket. However, as the entropy decreases, the returns and risks become more extreme, making our portfolio more volatile. We have also summarized the portfolio outcome, made by the three-month strategies as given in Table 2. The entropy values of AEM are undoubtedly larger than that of MVM, and hence the expected volatility of AEM is lower than that of MVM, though a certain expected return has undoubtedly been sacrificed. It is the decentralized investment that makes AEM behaving safer in either tumbling market situations. Currently, AEM can also be properly self-adjusted to make more fruitful investments.

To summarize, although the four strategies have similar systematic risks, AEM performs much better than EMWA in terms of excess returns due to the adaptive change in λ values. Obviously, AMVM performed very poorly, especially in 2014. Although AEM and MVM take the excess returns over each others, AEM turns out to be more diverse than MVM due to the entropy measures, which also meets the risk adverse preference for most investors. Owing also to the entropy measures, the maximum drawdown of AEM is consistently lower than that of MVM. If possible, under extreme market conditions, AEM is adaptive enough to outperform its MVM counterpart.

## 4. Conclusions

In this paper, we propose an adaptive entropy model (AEM), which incorporates factors including the expected return on the portfolio, the expected volatility, the entropy for the weights of the portfolio, and the adaptive λ values. We use data from the SSE50 index constituent stocks of five years of market data and compare our AEM results with Markowitz’s mean-variance model (MVM), the entropy model without adaptive effects (EMWA) and the adaptive mean-variance model (AMVM). From three perspectives of various portfolio performance indicators, the performance of AEM turns out to be better than the other existing models. In addition, in comparison to other models, the entropy and adaptability effects in AEM, make it more resilient to the rapidly changing market, while trying to maintain more decentralized investments. Last but not least, the existence and uniqueness of minimizers for AEM are briefly discussed.

## Figures and Tables

**Table 1:** Backtest Results.

Year	Model	α (%)	β	Annualized	Maximum	Calmar	Sharpe	Win
				Return (%)	Drawdown (%)	Ratio	Ratio	Rate (%)
2014	AEM	2.27	0.76	50.30	8.20	6.13	2.51	55.79
	EMWA	1.49	0.66	42.30	6.23	6.95	2.52	54.96
	AMVM	−18.1	0.8	17.24	33.83	0.51	0.53	52.8
	MVM	2.84	0.78	52.31	8.21	6.37	2.54	54.96
	Benchmark	0	1	63.58	9.88	6.44	2.85	50.83
2015	AEM	26.09	0.76	19.71	32.99	0.60	0.56	53.11
	EMWA	24.76	0.77	18.28	34.34	0.53	0.51	54.77
	AMVM	24.05	0.71	18.05	32.9	0.55	0.46	54.02
	MVM	11.24	0.78	4.70	39.10	0.12	0.13	53.94
	Benchmark	0	1	−8.41	44.66	−0.19	−0.21	51.04
2016	AEM	12.81	0.66	13.30	11.00	1.21	0.88	52.28
	EMWA	11.32	0.65	11.80	10.25	1.15	0.80	53.53
	AMVM	3.24	0.79	3.83	22.12	0.17	0.14	50.21
	MVM	14.58	0.65	15.06	9.05	1.66	0.98	52.30
	Benchmark	0	1	0.742	17.47	0.04	0.04	49.60
2017	AEM	16.15	0.65	32.16	6.10	5.27	3.04	57.44
	EMWA	10.92	0.63	26.30	6.08	4.33	2.53	56.61
	AMVM	20.17	0.89	42.08	16.38	2.57	1.58	59.09
	MVM	10.95	0.75	29.44	7.64	3.85	2.54	60.33
	Benchmark	0	1	24.60	6.38	3.86	2.22	53.72
2018	AEM	7.80	0.63	−5.77	20.57	−0.28	−0.33	49.80
	EMWA	6.80	0.65	−7.32	21.25	−0.34	−0.41	51.04
	AMVM	2.24	0.8	−15.19	33.71	−0.45	−0.47	50.21
	MVM	7.00	0.63	−6.70	22.07	−0.30	−0.37	49.80
	Benchmark	0	1	−21.70	28.46	−0.76	−1.00	49.80
2014–2018	AEM	14.72	0.72	20.6	33.14	0.62	0.96	53.74
	EMWA	11.88	0.71	17.74	34.43	0.52	0.83	54.31
	AMVM	10.67	0.8	17.24	33.83	0.51	0.53	52.8
	MVM	11	0.74	17.04	38.86	0.44	0.77	54.31
	Benchmark	0	1	8.2	44.7	0.18	0.33	51.15

Maximum Drawdown the lower the better while all other indicators the higher is the better.

**Table 2:** Three Month Portfolios for two models.

Date	Model	Expected-Returns	Expected-Volatilities	Entropy	λ Value
201508	AEM	−1.94	0.40	1.81	0
201508	MVM	−0.41	0.55	1.0	∖
201603	AEM	1.69	0.28	0.65	0.3
201603	MVM	1.72	0.30	0	∖
201708	AEM	2.40	0.46	0.63	0.6
201708	MVM	2.43	0.48	0.35	∖

## Abbreviations

The following abbreviations are used in this manuscript:

AEM	Adaptive Entropy Model
SSE50	Shanghai Stock Exchange 50
MVM	Markowitz’s Mean-Variance Model
EMWA	Entropy Model Without Adaptive Effects
AMVM	Adaptive Mean-Variance Model

## Appendix A

**Theorem** **A1.**
*The concavity of the entropy function.*

**Proof** **of Theorem A1.** According to the strictly concave definition, we set $P=(p_{1},p_{2},\cdots ,p_{n})$ and $Q=(q_{1},q_{2},\cdots ,q_{n})$ as two different probability distributions and take ω∈(0,1) to be a non-degenerate distribution. Hence, the proof of the strictly convexity of the information entropy, reduces to the following inequality

(A1) $$\omega H\left(P\right)+\left(1-\omega \right)H\left(Q\right)\leq H\left(\omega P+(1-\omega )Q\right),$$

then we get this result

(A2) $$-\omega \sum_{i=1}^{n}p_{i}\log p_{i}-\left(1-\omega \right)\sum_{i=1}^{n}q_{i}\log q_{i}\leq -\sum_{i=1}^{n}\left(\omega p_{i}+\left(1-\omega \right)q_{i}\right)\log\left(\omega p_{i}+\left(1-\omega \right)q_{i}\right),$$

which equivalent to

(A3) $$\omega \sum_{i=1}^{n}p_{i}\ln\frac{\omega p_{i}+\left(1-\omega \right)q_{i}}{p_{i}}+\left(1-\omega \right)\sum_{i=1}^{n}q_{i}\ln\frac{\omega p_{i}+\left(1-\omega \right)q_{i}}{q_{i}}\leq 0,$$

where the equal sign holds if and only if P=ωP+(1−ω)Q, which is P=Q. However, we have supposed P≠Q, So the equal sign in the above inequality does not hold. Since both ω and 1−ω are bigger than zero, we have

(A4) $$\sum_{i=1}^{n}q_{i}\ln\frac{\omega p_{i}+\left(1-\omega \right)q_{i}}{q_{i}}<0.$$

Hence, we have proved the theorem. □
